# Evaluating Disparities in Urban Versus Rural Mortality for Influenza and Pneumonia in the United States Using the CDC-WONDER Database Over a 22-Year Period

**DOI:** 10.7759/cureus.77475

**Published:** 2025-01-15

**Authors:** Oishi Barua, Nithin Karnan, Sumith Panayanthatta, Mohamed K Mansour, Chris Milano

**Affiliations:** 1 Gastroenterology and Hepatology, Children's National Hospital, Washington, D.C., USA; 2 Internal Medicine, K.A.P. Viswanathan Government Medical College, Tiruchirappalli, IND; 3 Clinical Research, Christian Medical College, Vellore, IND; 4 Hospital Medicine, Cleveland Clinic Abu Dhabi, Abu Dhabi, ARE; 5 Internal Medicine, American University of Integrative Sciences, Saint Michael, BRB

**Keywords:** cdc-wonder, influenza, metropolitan, pneumonia, retrospective, rural, urban

## Abstract

Introduction: The mortality of a disease alludes to the number of deaths caused by that disease within a specific population and timeframe. Urban-rural disparity indicates the differences in quality of life, access to healthcare and services, and economic opportunities between urban and rural areas. Thus, the study aims to evaluate the disparity in mortality rates between the urban versus rural areas for influenza and pneumonia over a 22-year period.

Methods: A retrospective study was conducted on 25^th^ May 2024, using the CDC-WONDER database, to analyze the mortality rates of influenza and pneumonia (ICD-10 codes J09-J18) based on rural and urban death, using the 2013 urbanization classification, and considering variables such as age, gender, and race.

Results: The absolute mortality in rural areas (511,454 deaths; 19.3%) is approximately four times lower than urban areas (2,002,659 deaths; 79.7%).The trend in mortality rate per 100,000 has gradually decreased over the years. The mortality rate per 100,000 population based on age, gender and race stratification is significantly higher in rural areas compared to the urban areas.

Conclusion: This study highlights the significant disparities in urban versus rural mortality for patients with influenza and pneumonia, particularly in the 85+ years age group, female gender, and White American race.

## Introduction

The influenza virus is an RNA virus belonging to the *Orthomyxoviridae *family and is classified into types A, B, and C based on its nucleoprotein and matrix protein composition [1]. Influenza causes an acute respiratory illness and can affect individuals of all ages worldwide [2]. It can result in outbreaks and epidemics particularly in the winter season [2]. Based on the Centre for Disease Control and Prevention (CDC)'s previous estimates of the influenza disease burden, it is estimated that in the United States, the influenza virus resulted in 9.3 to 45 million illnesses and 12000 to 61000 deaths during the annual influenza epidemics, from the years 2010 to 2020 [2]. These figures highlight the importance of recognizing the influenza virus infection as an important public health problem and working on measures to prevent and treat it, particularly given the disparities between rural and urban areas with respect to this disease condition.

Geographic disparities in health and disease have been an important area of public health research in the United States [3]. Reduction of health disparities between rural and urban areas has been an important public health policy goal since 1990 and is an important part of the CDC’s health equity strategy [3,4]. Understanding the magnitude and reasons for mortality differences between rural and urban areas is important for the purposes of public health decision-making and social planning [3,5]. This analysis of health disparities for certain disease conditions is important, as it allows the allocation of public health resources towards those in rural or urban areas, who are at risk of mortality from certain disease conditions [3].

It is hypothesized that mortality rates for influenza and pneumonia would be higher in rural areas due to disparities in access to healthcare and preventative measures, such as vaccination The paucity of research examining the disparities in mortality rates of influenza and pneumonia between rural and urban geographic areas in the United States prompted us to undertake this study.

Aims and objectives

The primary objective of this study is to analyze the differences in crude mortality rates between urban and rural areas in the United States over a 22-years, utilizing data from the CDC-WONDER database. The secondary objectives examine the variations in mortality rates based on factors such as age, gender, and race.

## Materials and methods

The data used in this retrospective analysis were collected on May 25, 2024, from the CDC Wide-ranging Online Data for Epidemiologic Research (CDC-WONDER) platform. Ethics committee approval was not sought as this data is deidentified and publicly available. The "Underlying Causes of Death" dataset was selected from the CDC-WONDER homepage, and the time frame was specified for the years 1999 to 2020. In order to focus on influenza and pneumonia-related mortality, the "Underlying Cause of Death by Bridged-Race Categories" module was accessed.

ICD-10 codes J09 to J18, which correspond to influenza and pneumonia, were selected as the underlying cause of death for the analysis. The dataset was stratified by key demographic variables such as age, gender, race, and geographic location, allowing for the examination of trends and mortality rates across these categories. The Metropolitan 2013 classification categorizes urban/metropolitan areas into four populations: large central metropolitan, large fringe metropolitan, medium metropolitan, and small metropolitan. Rural non-metropolitan areas were subdivided into micropolitan and non-core. The mortality rate for each census region was extracted.

The secondary objective was to determine mortality rates by age, gender, and race, with age categorized in 10-year ranges. All data were extracted separately.

The data was then exported to Microsoft Excel and statistical analysis was done using RStudio v4.3.2, and plots were created using the GGPlot 2 package. The analysis included the usage of binomial tests to determine the association between demographic variables and mortality rates. P-value <0.05 was considered to be statistically significant.

## Results

Using the CDC-WONDER database, an aggregate total of 2,514,114 deaths were reported for influenza and pneumonia from 1999 to 2020. The absolute number of deaths due to pneumonia and influenza in urban areas is 79.7% (n= 2,002,659) and 19.3% (n= 511,454) in rural areas from 1999 to 2020 (Table 1).

**Table 1:** Absolute number of reported mortalities in urban and rural areas due to influenza and pneumonia from 1999-2020 as per the 2013 Urbanisation Classification

Type	n (%)
Urban (Metropolitan area)	2,002,659 (79.7%)
Large Central Metropolitan	717,034 (35.8%)
Large Fringe Metropolitan	537,298 (26.8%)
Medium Metropolitan	503,361 (25.1%)
Small Metropolitan	244,966 (12.2%)
Rural (non-metropolitan area)	511,454 (19.3%)
Micropolitan	277,509 (54.3%)
Non-core	233,945 (45.7%)

**Figure 1:**
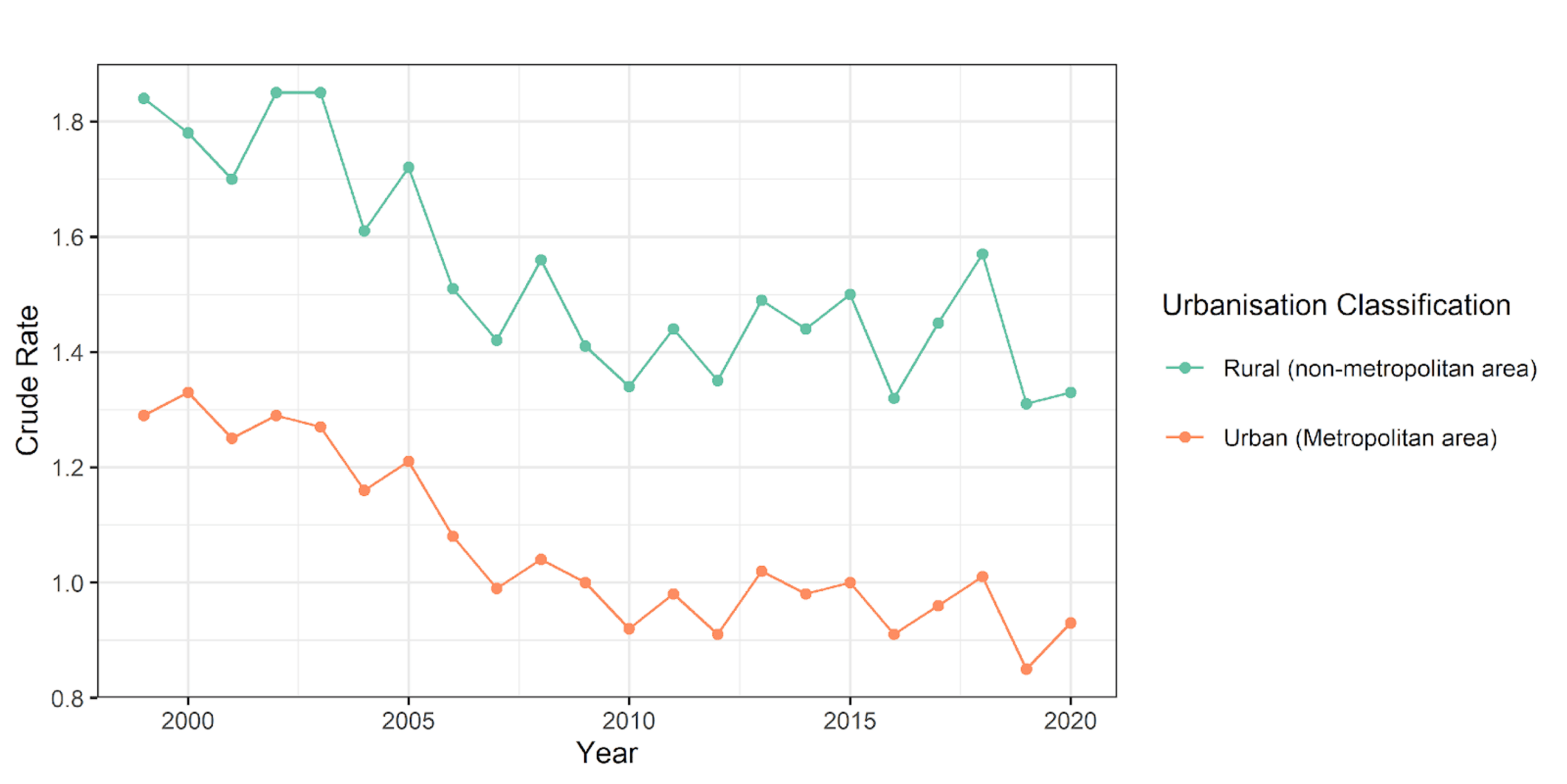
Line diagram showing trends in urban versus rural mortality due to influenza and pneumonia from calculated in mortality rate per 100,000 population

Figure 1 shows the urban and rural mortality trends due to influenza and pneumonia using the mortality rate per 100,000 population. The overall mortality rates are declining except for the years 2003, 2005, 2007, and 2017, when the mortality rate was slightly higher compared to the previous years.

Table 2 shows the mortality rate per 100,000 due to influenza and pneumonia in urban and rural areas based on age, gender, and race.

**Table 2:** Mortality due to influenza and pneumonia in urban and rural areas based on age, gender and race

Variables	Urban			Rural			Binomial Test
	Mortality	Total population	Mortality Rate (per 100,000)	Mortality	Total population	Mortality Rate (per 100,000)	P value
Age Groups							
< 1 year	3784	2394185312	0.16	734	387051552	0.19	<0.001*
1-4 years	1862	9585972416	0.02	307	1568264800	0.02	<0.001*
5-14 years	1768	24644041056	0.01	292	4195063744	0.01	<0.001*
15-24 years	3352	25592179680	0.01	673	4353219360	0.02	<0.001*
25-34 years	7401	25697740000	0.03	1668	3745083488	0.04	<0.001*
35-44 years	16611	25725321728	0.06	3604	4075832960	0.09	<0.001*
45-54 years	37688	25235508448	0.15	8559	4446895936	0.19	<0.001*
55-64 years	71471	20452379392	0.35	17394	4073167520	0.43	<0.001*
65-74 years	126984	13375621664	0.95	32276	2959011936	1.09	<0.001*
75-84 years	273442	7813886368	3.5	69601	1738244608	4	<0.001*
85+ years	455209	3146384704	14.47	119620	678056960	17.64	<0.001*
Gender							
Male	465722	90081775680	0.52	116895	16073356800	0.73	<0.001*
Female	535531	93581445088	0.57	138758	16146536064	0.86	<0.001*
Race							
American Indian or Alaska Native	3623	2008024800	0.18	3970	819533312	0.48	<0.001*
Asian or Pacific Islander	34652	11519147712	0.3	1150	382082848	0.3	<0.001*
Black or African American	107800	26584539232	0.41	15885	2824575808	0.56	<0.001*
White	854747	1.43552E+11	0.6	234482	28193700896	0.83	<0.001*

The age group that has the highest mortality rate per 100,000 in both urban and rural areas is above 85 years. The mortality rate was observed to be significantly higher in rural areas (17.64) as compared to urban areas (14.47). Based on gender, the female gender has a higher crude rate and the mortality rate was higher in rural areas (0.86) as compared to urban areas (0.57). In terms of race, the crude rate was higher in the White race and the mortality rate was higher in rural areas (0.86) than in urban areas (0.6).

Although the absolute number of mortalities was higher in urban areas compared to the rural areas (Table 1), the mortality rate per 100,000 population based on age, gender, and race stratification is significantly higher in rural areas compared to the urban areas (Table 2).

## Discussion

An original retrospective study was conducted to evaluate the discrepancy in the mortality trend of pneumonia and influenza in the USA over 22 years from 1999-2020. This study disclosed that the absolute number of mortalities from influenza and pneumonia was higher in the urban areas compared to the rural areas. The mortality rate per 100,000 population based on age, gender, and race stratification is significantly higher in rural areas compared to urban areas.

It is crucial to study the differences and magnitude of mortality trends among the rural and urban populations to address the public health disparities in these areas. It also provides effective social planning and policies to bring down the mortality rates [7,8]. The reasons for such diversion seem to be multifactorial: socioeconomic status, race/ethnicity, modifiable lifestyle behavior, etc [9]. In this study, the mortality rate per 100,000 population based on age, gender, and race was higher in rural areas compared to urban areas. This signifies that rural areas have limited access to advanced healthcare services and sophisticated technologies making this a critical concern for healthcare policymakers which needs to be meticulously evaluated to support and nurture the health equity of the rural communities [10,11].

Ashraf et al. reported that rural areas had a consistently higher mortality rate for pneumonia and influenza in the United States, which coincides with our findings. They believe that vaccination is an important determinant for such discrepancies among the rural population [12]. Also, effective interventions and preventive measures towards the high-risk residents can successfully reduce such variation in the numbers between the two communities.

Research has suggested that female patients may face a higher risk of fatal outcomes from pneumonia, which is consistent with the trends observed in our study [13]. This increased vulnerability may be attributed to various factors, including hormonal differences, comorbid conditions, and access to healthcare. Another study has highlighted a significant disparity in pneumonia mortality rates, revealing that Black Africans experience higher mortality rates compared to their White counterparts [14]. This finding contradicts our observation and thus raises important questions about underlying social determinants of health, including access to healthcare, socioeconomic factors, and potential differences in disease presentation or treatment responses between these populations.

More research is needed to address this growing issue to focus on the exact causes of disparity. Adequate healthcare facilities and the availability of appropriate management for rural people who face discrimination in some domains of access to healthcare should be ensured [15]. Influenza and pneumonia-related complications, such as pneumonia complicated by sepsis, respiratory failure, secondary infections, etc., should be properly managed in tertiary healthcare facilities. The COVID-19 pandemic and rural hospital closures could further exacerbate the trend [16]. Therefore, standardized protocols should be implemented for faster diagnosis in the community clinics and to avoid delays in referrals and transportation to the tertiary hospitals for management. This will not only directly reduce the rural mortality rates of pneumonia and influenza but also indirectly reduce the mortality trends of the transferred patients in urban hospitals.

Limitations

The CDC-WONDER database does not include data from during the COVID-19 pandemic (2021-2023). This study did not classify pneumonia and influenza based on its sub-categories as data related to the causes of death specific to pneumonia and influenza is not listed in the CDC-WONDER database. Likewise, modifiable factors like socioeconomic causes and access to healthcare could not be studied.

## Conclusions

This study highlights notable disparities in mortality rates due to pneumonia and influenza between urban and rural areas. The absolute number of reported mortalities in urban areas is higher compared to rural areas. However, the mortality rate (per 100,000 population) is higher in rural areas compared to urban areas. The mortality rate shows a declining trend. Demographically, the highest mortality rates are observed in individuals aged 84 and older, particularly among females and those identifying as White.

Effective interventions should target these vulnerable groups, and further research is essential to understand the specific reasons behind these disparities. More research studies should be undertaken to explore the specific reasons for such a discrepancy and constructive policies need to be introduced by the policymakers aimed at reducing mortality rates. Further research is needed to understand the interplay between these factors and to develop targeted interventions that address both gender and racial disparities in pneumonia outcomes.
